# Gender Inequality in Household Chores and Work-Family Conflict

**DOI:** 10.3389/fpsyg.2018.01330

**Published:** 2018-08-03

**Authors:** Javier Cerrato, Eva Cifre

**Affiliations:** ^1^Department of Social Psychology, Faculty of Labour Relations and Social Work, Universidad del País Vasco (UPV/EHU), Bilbao, Spain; ^2^Department of Developmental, Educational and Social Psychology and Methodology, Universitat Jaume I, Castellón de la Plana, Spain

**Keywords:** gender inequality, work-family conflict, households, organizations, Gen Xers

## Abstract

The fact that the permeability between family and work scopes produces work-family conflict (WFC) is well established. As such, this research aims to check whether the unequal involvement in household chores between men and women is associated with increased WFC in women and men, interpreting the results also from the knowledge that arise from gender studies. A correlational study was carried out by means a questionnaire applied to 515 subjects (63% men) of two independent samples of Spanish men and women without emotional relationship, who lived with their heterosexual partner. As expected, results firstly show unequal involvement in household chores by women and men as it is higher in women that in men, and the perception of partner involvement is lower in women that in men. Secondly, those unequal involvements relate differently to men and women on different ways of work-family interaction. They do not increase WFC in women comparing to men, although there are tangentially significant differences in work conflict (WC) and statistically significant in family conflict (FC). However, perception of partner involvement on household chores increases WFC both in men and in women but not WC nor FC. Nevertheless, increase on marital conflict (MC) by domestic tasks neither affect in a significant way WFC in women nor in men, but increase WC in both women and men and FC only in women. Results also confirm that subject involvement on household chores is not a significant predictor of WFC in women nor in men, and that MC by domestic tasks is a statistically significant predictor in women of WFC and FC, but not in men. Thus, results show that traditional gender roles still affect the way men and women manage the work and family interaction, although the increased WFC due to involvement in housework is not exclusive to women, but also occurs in men. Personal and institutional recommendations are made on the basis of these results to cope with these conflicts.

## Introduction

Occupational health psychology promotes labor risk prevention intervening both on the organization and on the person, but also on work-family interface. It seeks the goodness-of-fit among these dimensions in order to reduce psychosocial risks on occupational health and concurrently to improve organizational efficacy. The effect of psychosocial stressors at work does not remain within the working sphere as it extends also to personal life. This permeability between family and work scopes has produced work-family conflict (WFC) to be one of the psychosocial risks receiving more attention during the past years (Eby et al., 2005; Ammons and Kelly, 2015; French et al., 2017; Lapierre et al., 2017; Wayne et al., 2017; Carvalho et al., 2018). WFC negatively affects both health and general life such as work performance and work satisfaction within the organizational context, but it also increases conflict rates and decreases family satisfaction. From this perspective, and within a context of a more technological and digitalized society, gender equality at work is a matter of paramount importance, which must start with a gender equality at home. The aim of this study is to check whether the unequal involvement in household chores between men and women is associated with increased WFC in women, and explain it in terms integrating the knowledge of gender studies.

### Work-Home Conflict and Gender

Individuals may experience conflict between their work and home roles due to limited time, high levels of stress, and competing behavioral expectations (Greenhaus and Beutell, 1985). Although most of the work-home research has focused on how work variables affect home from the point of view of the conflict between the two spheres (Major and Cleveland, 2005), organizational psychology also begins to study how family variables affect job performance and satisfaction.

In the psychosocial scientific literature, there is a wide tradition on the work and home interface studies (i.e., Kopelmanś et al., 1983; Edwards and Rothbard, 2000; Pitt-Catsouphes et al., 2006; Mills, 2015; Paulin et al., 2017). Two primary perspectives have been offered in this literature based on the incompatibility between individuals’ work and home domains (Michel and Hargis, 2008). One perspective focuses on the mechanisms that generate conflict between both domains. The other perspective focuses on the segmentation mechanisms between the work and the family domains. In this study, we adopt the conflict model in examining the influence of home roles (differential involvement of men and women on household chores), on work roles.

Some research has shown that role pressure in work and home domains generates negative consequences on the other one bidirectionally. So the degree of participation in the home role will create difficulties for participation in work, resulting in the home-work conflict (HWC); conversely, the degree of participation in the work domain can hinder performance on the family role, producing an increase of strain-based, time-based or behavior-based work-home conflict (WHC) (Huang et al., 2004).

Gender roles are essential for understanding the work-home interface. They are shared beliefs that apply to individuals on the basis of their socially identified sex which are the basis of the division of labor in most societies (Wood and Eagly, 2010). In Western societies, the home sphere, and the household chores as part of this sphere, it is assumed to be in charge of women, which could in turn affect more highly the home to work conflict of women than of men. However, to our knowledge, this has not been checked empirically. In this study we will focus on the effect of the relationship between gender and dedication to household chores on WFC among women.

Different meta-analyses (Byron, 2005; Eby et al., 2005) have demonstrated the key role played by gender, but how it relates to work-family constructs is still both theoretically and empirically debated (Shockley et al., 2017). Research has found differences in work-home conflict repeatedly, ranging from differences in the experience of WFC to the existence of different work and home backgrounds to women and men. However, most studies in the field of work-home interface do not consider gender as a variable, identifying at most correlates and differential associations for men and women (Martínez and Paterna, 2009). Thus, we posit that work-home interface studies should include gender as key variable due to the influence of gender ideology and gender-role orientation might have on the work-home relationship from a cultural point of view.

From a cultural and discursive perspective (Gerstel and Sarkisian, 2006), gender ideology, defined as beliefs and values maintained about what is right for men and women, determines the patterns by which a particular society judges or evaluates the proper conduct of a man or a woman.

This gender ideology is also reflected in the social discourse, as frequently the couple recreates the dominant social discourse in which is referred the essential characteristics in which men and women differ ignoring the sociopolitical context. This discourse states that the differences between men and women in relation to home and work are the result of personal choice, that there are differences in innate abilities of men and women for household chores and work outside the home, and that these differences guide the choice for certain jobs and even that preference for home toward work is a free choice in the case of women (Martínez and Paterna, 2009; Kuo et al., 2018). Linked to this ideology, the traditional gender role model prescribes that work domain and instrumentality are more important for men than for women, whereas the home domain and expressiveness is more important for women. The traditional gender role model has a biosocial and cultural origin, and was described by Parsons and Bales (1955) in their delineation of instrumental (men) and expressive (women) roles. This model arbitrarily assumes that expressiveness and instrumentality are separate dimensions, and that expressiveness is always women gender role whereas instrumentality is that of men. Work and family interactions are embedded in the broader cultural, institutional and economic context in which individuals reside (Ollier-Malaterre and Foucreault, 2017). Of particular relevance to gender differences in WFC are cultural differences in gender egalitarianism, or belief or attitudes about de equality of the sexes within de culture (House et al., 2004; Lucas-Thompson and Goldberg, 2015).

As Martínez and Paterna (2009) indicate, gender ideology seems to determine the percentage of tasks considered traditionally feminine by members of the couple, such as washing, ironing, shopping, cooking, or cleaning. It also generates a differential meaning about household chores for men and women. Also, recent studies have shown that there is still a division of house chores by gender, depending on the gender role nuclei: instrumentality inside and outside home for men; expressiveness and instrumentality inside home for women (Fernández et al., 2016). All this rationale, leads us to formulate hypothesis 1:

H1: There will be a division of household chores between men and women based on traditional gender roles. Women will spend more time than men in traditionally female household chores and men in traditionally male ones.

Both men and women similarly perceive a lack of parity in performing household chores, but perceive greater equality in the care of daughters and sons (Yago and Martínez, 2009). This leads us to propose hypothesis 2:

*H2*: *Women will perceive their partners much less involved in household chores and only focus on household chores traditionally considered masculine. Men will perceive their female partners more involved in traditionally female household chores, especially in those traditionally considered feminine.*

### Implication in Household Chores and Work-Family Conflict (WFC)

Time required for household chores and caring for the family is one of the most important factors in the conflict coming from the family sphere, especially in families with children. So, the dual-income couples with children tend to have a greater number of conflicts between the partners and a higher level of stress than their counterparts without children (Michel and Hargis, 2008). From this point of view, the gender roles model assumes that the nature of the role demands differs in men and women, and these roles act as moderators of WFC (Barnett et al., 1995).

The highest level of family to work interference in women comes from the different implication of women and men in household chores, including the care of children. This different implication has been proven by various studies and research (Bianchi et al., 2000; Korabik, 2015; Borelli et al., 2017) and still persists in society as has been found in different surveys (Organization for Economic Cooperation and Development [OECD], 2014; Eurobarometer, 2015). In concrete, this model keeps very persistent in Spain, where women spend almost double the amount of time on unpaid work as men National Institute of Statistics (INE), 2018). This time is spent on activities such as caring for children (38 h a week women versus 23 men) or family members (20 h women versus 14 men) or household chores (20 h women versus 11 men). So although women have begun to strongly form part of the labor force and to spend more time with their children taking care of them, they neither assume a decrease in their salary as much as women do for work interruptions due to family issues nor stay at home to take care of their children (Gerstel and Sarkisian, 2006). Most men still maintain full involvement in their work because their feminine couple assume the responsibility for caring their children. Thus, we can deduce that women will suffer more by the interference of the family at work, because their greater involvement in the family will can subtract them time, strength and dedication to their work; however, men will suffer more by the interference of work in the family. In fact, a high implication in the family sphere has been shown linked to a higher family-to-work interference only in women (Hammer et al., 1997).

Moreover, men do not feel an obligation when they are involved in the home as women do, as they perceive it more as a hobby or a free choice. Also, those house chores that keep the home every day (shopping, cooking, washing dishes, washing clothes, and cleaning the house) are considered feminine, while those considered male or neutral tasks (paying bills, taking care of the car or home maintenance) do not involve daily devotion. Some cultural interpretation argue that women are more involved in house chores and do not want to fully share because of the belief that this is central to their gender identity and a source of power in the family, whereas husbands, whose gender identity has traditionally been marked by paid work, would not object to do less household chores than their wives (Martínez and Paterna, 2009).

Besides, a crossover effect must be included: to the greater involvement of women in the family and household chores must be added the greatest involvement of men in the workplace (Bakker et al., 2008), which supposes an increased family burden for women. As husbands are not available for household chores, wives suffer overload by household chores and emotional demands related to children caregiving, which will increase still more women stress and family to work interference (Frone, 2003).

In short, the lesser involvement of men in household chores and greater transfer of stress from work to family causes increased domestic workload on women and marital conflict (MC), thus increasing the tension transfer from family environment to worksite in women. All this rationale, leads us to formulate hypothesis 3:

H3: The greater involvement of women in household chores and the perception of the lesser involvement of their men partners is linked to an increased family to work conflict (FWC) in women.

### Marital Conflict and Household Chores

This greater involvement of women in household chorus and increased family to work conflict may lead to an increase of MC. In this line, Pittman et al. (1996) provide evidence for this idea by showing that the contribution of women to household chores is higher on the days when their husbands express higher levels of work stress; in these cases, women must subtract energy and time from work due to their husbands’ increased work stress. However, men do not adjust their contribution to household chores when their wives bring their work stress home. Research on family processes shows that stressed couples show a high level of negative interactions and conflicts. Thus, increased stress associated with WFC and its correlative frustration, leads individuals to initiate or exacerbate their sequence of negative interaction with the partner (Westman and Etzion, 2005; Huffman et al., 2017). This negative interaction may be understood as product both of *social undermining* which consist in behaviors that involve rejection, criticism and negative attitude toward the couple (Vinokur and Van Ryn, 1993) and *hostile marital interactions* (Matthews et al., 1996), which aims to express hostility toward the partner or MCs.

Focusing on the conflict between the partners and their relationship with household chores, it has shown how increasing distress and frustration generated by the WFC tends to impair the interaction with the partner (Westman and Etzion, 2005). This can result in increased tension between the partners due to the transfer of stress from work to family by men and their lesser involvement in household chores, which would generate an increase in MC and, therefore, an increase of conflict in the family especially in women due to unequal distribution of household chores. This leads us to propose hypothesis 4:

H4: The conflict between the partners due to unequal distribution of household chores generates an increase of more family to work conflict (FWC) in women than in men because of their greater involvement at home.

## Materials and Methods

### Participants and Procedure

A correlational study was carried out by means of a questionnaire applied by professional surveyors during 2014. They selected a segmented sample of men and women working in public and private organizations from different productive sectors (teaching, services, and manufacturing sectors). The final sample consisted of 515 subjects, mostly (63%) were men, with an average age of 40 years old; all of them were married or living with a heterosexual partner, and they had children. Samples of men and women were independent from each other, without emotional/marital relationship between them. Regarding the organizational setting, 21% were working in public organizations and 79% in private ones.

### Measures

• *Work-Family Conflict (WFC), Family Conflict (FC), and Work Conflict (WC)* based on time and strain were measured through the Spanish version (Martínez-Pérez and Osca, 2001) of the Kopelmanś et al. (1983) scale. This scale applies the role conflict concept of Kahn et al. (1964) to study work and family scopes first separately and then together, based on the idea that WC and FC might act as antecedents of WFC. Each of these subscales consists of eight items on a Likert scale ranging from 1 (total disagree) to 5 (total agree). An example of a *WFC subscale* item is *My work timetable is often incompatible with my family life*; an example of an item from the *FC subscale* is *My family dislikes doing some activities I would like to do;* and an example of an item from the *WF* subscale is *At work I can’t be myself, or be the way I really am.*
• *Subject involvement with household chores* scale. This is a 10-item self-constructed scale that measures subjects’ self-perception about different tasks related to household chores, family management, and child care and education. Subjects respond to each item using a dichotomous yes/no format. The final scale score is the total number of family tasks they do. Examples of these items are *Do you take the children to school every day?* and *Do you clean your house in your everyday life?* This scale only includes the most common household chores of a standard Spanish couple with children of school age, not including others that may be less frequent in this culture (i.e., cutting the grass).
• *Partner involvement in household chores perception scale*. This self-constructed scale is similar to the one above, but in this case it measures the subjects’ perception of their partners’ involvement in all the household chores. Subjects respond to each item using a dichotomous yes/no format about their perception of their partner’s involvement in different family tasks. The final scale score is the total number of tasks they perceive that their partners dedicate to family tasks. An example of these items is *Does your partner take the children to school in everyday life?*
• *Marital conflict* *about household chores* was measured with the single question *How many times do you and your partner argue about who must do the household chore*s *and when*? Subjects respond to this item on a Likert scale ranging from 1 (never) to 5 (every day).

We also measured socio-demographic (sex and age) and socio-familiar (family status, number of children) variables for the sample description.

### Data Analyses

First, we performed skewness and kurtosis analyses to check normality among variables. Second, we calculated internal consistencies (Cronbach’s α), descriptive analyses and correlations between conflict scales and subject/partner perceived involvement on household chores scales. Third, we computed Analyses of Variance (ANOVAs) in order to test whether there was any statistically significant difference between-group regarding gender for subject’s involvement in household chores scale, and subject’s perception of partner’s involvement in household scale, and Kruskal–Wallis non-parametrical tests for item to item analysis due to its dichotomous level of response (Hypothesis 1 and 2). After that, we computed new ANOVAs and Regression Analyses to check gender, household chores, partner’s implication and conflict on WFC, WC, and FC (Hypothesis 3 and 4). All data analyses were carried out using SPSS 21.0.

## Results

**Table 1** shows skewness and kurtosis statistics. As expected, all scales show values equal or below 0.5 and −0.5 in both or at least at one of them. So we assume a normal distribution of the scores of these scales. However, item by item of subject’s and partner’s involvement in household chores scales do not follow that normal distribution, due to its dichotomical nature.

**Table 1:** Skewness and Kurtosis analysis of variables distribution.

	Skewness		Kurtosis	
	Statistics	*SE*	Statistics	*SE*
Work-family conflict	−0.02	0.11	−0.27	0.22
Work conflict	−0.20	0.11	−0.50	0.22
Family conflict	0.56	0.11	−0.16	0.22
Marital conflict	0.46	0.11	−0.80	0.22
**Involvement on household chores (total mean scale score)**				
Subject involvement on household chores	0.79	0.15	0.01	0.30
Perception of partner involvement on household chores	0.76	0.15	0.31	0.30
**Subject involvement on household chores (item to item)**				
Home shopping	0.92	0.11	−1.16	0.21
Cleaning home	0.38	0.11	−1.87	0.21
Domestic repairing	0.84	0.11	−1.30	0.21
Family management	0.69	0.11	−1.53	0.21
Free time family management	1.62	0.11	0.63	0.21
Take children from home to school	1.14	0.11	−0.71	0.21
Take children from school to home	1.19	0.11	−0.60	0.21
Children caregiving	0.73	0.11	−1.47	0.21
Helping children with homework	1.20	0.11	−0.57	0.21
Playing with children	3.60	0.11	10.85	0.21
**Perception of partner involvement on household chores (item to item)**				
Home shopping	2.1	0.11	2.37	0.21
Cleaning home	1.4	0.11	0.02	0.21
Domestic repairing	0.33	0.11	−1.90	0.21
Family management	1.22	0.11	−0.51	0.21
Free time family management	2.66	0.11	5.11	0.21
Take children from home to school	1.57	0.11	0.47	0.21
Take children from school to home	1.49	0.11	0.24	0.21
Child caregiving	1.97	0.11	1.92	0.21
Helping children with homework	2.13	0.11	2.58	0.21
Playing with children	4.43	0.11	17.70	0.21

**Table 2** shows the descriptive analyses and Cronbach’s alpha of the variables for both samples. The alpha values meet the criterion of 0.70 (Nunnally and Bernstein, 1994), except in the case of the perception of partner’s involvement in household chores, which was above 0.60. As expected, the pattern of correlations shows that WFC, work conflict and FC are positively and significantly related in both samples. However, WFC is more related to conflict at work in women and to conflict in the family in men.

**Table 2:** Cronbach’s alpha, means (*M*), standard deviation (*SD*), and intercorrelations by gender (*N* = 515).

		Women							Men						
	α	*M*	*SD*	1	2	3	4	5	*M*	*SD*	1	2	3	4	5
(1) Work-family conflict	0.78	2.1	0.70						2.0	0.70					
(2) Work conflict	0.78	2.7	0.75	0.34^∗∗^					2.9	0.87	0.28^∗∗^				
(3) Family conflict	0.76	2.4	0.81	0.26^∗∗^	0.30^∗∗^				2.3	0.87	0.33^∗∗^	0.40^∗∗^			
(4) Marital conflict	–	2.4	1.3	0.31^∗∗^	0.21^∗∗^	0.42^∗∗^			2.2	1.0	−0.05	0.10	0.03		
(5) Subject involvement on household chores	0.72	4.0	0.15	−0.17^∗^	−0.16	0.09	0.12		1.7	0.12	−0.18^∗∗^	−0.23^∗∗^	0.18	0.08	
(6) Perception of partner involvement on household chores	0.62	1.8	0.98	0.14	0.14	0.04	−0.18	−0.49^∗∗^	2.8	1.5	0.31^∗∗^	0.02	0.08	−0.10	−0.13

*^∗∗^p < 0.01, ^∗^p < 0.05.*

Marital conflict is only highly and positively related to WFC, work conflict and FC in women, but not in men. This could indicate that women assimilate the conflict with the partner into conflicts in the family, i.e., women integrate the couple into the family concept, while men consider them to be different.

Subject’s involvement in household chores correlates significant and negatively with WFC in both men and women, but only with work conflict in men. Then, for both men and women, the higher their involvement is in household chores, the lower their WFC; moreover, the higher the work conflict is, the lower the men’s involvement in household chores.

Finally, the correlation between the subject’s and the perception of the partner’s involvement in household chores is only highly, significantly and negatively related in women. However, the perception of the partner’s involvement in household chores is only highly, significantly and positively related to WFC in men. Thus, women decrease their involvement in household chores when their male partners increase their involvement; on the other hand, in the case of men, the greater the involvement of the partner (women) in the household chores, the higher the WFC is.

ANOVA results confirm these differences and inequality about men’s and women’s involvement in household chores. Women’s involvement in household chores is more than twice that of men (4.0 and 1.7, respectively; *F* = 82.60; *p* ≤ 001). Consistently, women perceive lower involvement of their partner (men) in household chores than men do (1.8 and 2.8, respectively; *F* = 22.70; *p* ≤ 001).

Kruskal–Wallis tests also confirm that women are significantly more involved than men in seven of eleven household chores (see **Table 3**). These seven tasks are traditionally considered feminine: home shopping, house cleaning, free-time family management, taking children from home to school and from school to home, children’s care, helping children with homework, and playing with them. Men only score higher than women on one task traditionally considered masculine: house repairs. There are no differences in family management. These results confirm Hypothesis 1.

**Table 3:** Kruskal–Wallis test of subject involvement on household chores and perception of partner involvement on household chores by gender (item to item) (*N* = 515).

	Women		Men			
	*M*	*SD*	*M*	*SD*	Chi-square	GL
**Subject involvement on household chores (item to item)**						
Home shopping	**0.40**	0.49	0.13	0.33	39.078^∗∗∗^	1
Cleaning home	**0.61**	0.49	0.05	0.22	153.846^∗∗∗^	1
Domestic repairing	0.10	0.30	**0.67**	0.47	180.924^∗∗∗^	1
Family management	0.31	0.47	0.38	0.49	2.455	1
Free time family management	**0.22**	0.41	0.13	0.33	6.725^∗∗^	1
Take children from home to school	**0.32**	0.47	0.13	0.34	22.959^∗∗∗^	1
Take children from school to home	**0.34**	0.47	0.08	0.28	41.483^∗∗∗^	1
Children caregiving	**0.50**	0.50	0.04	0.20	109.332^∗∗∗^	1
Helping children with homework	**0.35**	0.48	0.07	0.25	49.258^∗∗∗^	1
Playing with children	**0.10**	0.28	0.03	0.16	6.923^∗∗^	1
**Perception of partner involvement on household chores (item to item)**						
Home shopping	0.08	0.28	**0.24**	0.43	24.355^∗∗∗^	1
Cleaning home	0.05	0.22	**0.50**	0.50	141.873^∗∗∗^	1
Domestic repairing	**0.64**	0.48	0.03	0.16	187.264^∗∗∗^	1
Family management	**0.30**	0.46	0.14	0.35	15.260^∗∗∗^	1
Free time family management	0.07	0.26	**0.15**	0.36	7.49^∗∗^	1
Take children from home to school	0.12	0.33	**0.30**	0.46	24.446^∗∗∗^	1
Take children from school to home	0.10	0.31	**0.37**	0.48	51.522^∗∗∗^	1
Child caregiving	0.05	0.22	**0.32**	0.47	66.873^∗∗∗^	1
Helping children with homework	0.08	0.28	**0.23**	0.42	21.669^∗∗∗^	1
Playing with children	0.04	0.20	0.04	0.20	0.000	1

*The highest values, when significant, appear in bold. ^∗∗∗^p ≤ 001, ^∗∗^p ≤ 01.*

Symmetrically, Kruskal–Wallis tests also show that these results are confirmed by the perception that men and women have of their partner’s involvement in household chores: men consider that their partners (women) are mainly involved in traditionally feminine household chores: home shopping, house cleaning, free-time family management, taking children from home to school and school to home, taking care of the children, and helping children with homework, whereas women consider that their partners (men) are involved in typically masculine household chores: house repairs and family management. There are no differences in the perception of playing with the children. On the whole, these results confirm Hypothesis 2.

To test the hypothesis 3 (the effect of the greater involvement of women in household chores and perception of lesser involvement of male partners in the increase in the WFC among women compared to men), and hypothesis 4 (the effect of MC in the increased level of WFC in women relative to men), we performed three separate ANOVAs (**Table 4**), complemented by multiple regression analysis (**Table 5**).

**Table 4:** Analysis of variance of work-family conflict, work conflict and family conflict by subject involvement on household chores and subject perception of partner involvement on household chores and marital conflict by gender (*N* = 515).

	Gender	Work conflict		Family conflict		Work-family conflict	
		*M*	*SD*	*M*	*SD*	*M*	*SD*
**Subject involvement on household chores**							
High	Women	2.7	0.98	2.8	1.0	2.2	0.85
	Men	3.9	0.27	3.1	0.85	2.0	0.87
Low	Women	2.9	0.69	2.6	0.80	2.4	0.72
	Men	3.2	0.90	2.1	0.92	2.3	0.71
	*F*	2.516^+^	3.552^∗^	1.204
**Perception of partner involvement on household chores**							
High	Women	2.9	0.35	2.9	1.1	2.7	0.53
	Men	3.8	0.53	2.6	0.92	2.4	0.66
Low	Women	2.6	0.84	2.5	0.90	2.0	0.77
	Men	3.3	0.93	2.6	0.95	1.8	0.68
	*F*	2.330	0.690	8.458^∗∗∗^
**Marital conflict**							
High	Women	2.9	0.77	3.0	0.80	2.3	0.74
	Men	3.8	0.85	2.6	0.71	2.1	0.54
Low	Women	2.7	0.76	2.4	0.70	2.3	0.67
	Men	3.4	0.89	2.6	1.0	2.2	0.78
	*F*	3.273^∗∗^	7.442^∗∗^	0.533

*^∗∗∗^p ≤ 0.001, ^∗∗^p ≤ 0.01, ^∗^p ≤ 0.05, ^+^p ≤ 0.10.*

**Table 5:** Regression analyses predicting work conflict, family conflict and work-family conflict (dependent variables) in women and men by involvement on household chores, subject perception of partner involvement on household chores and level of marital conflict (independent variables).

	Work Conflict		Family Conflict		Work-Family Conflict	
	Women	Men	Women	Men	Women	Men
	
	β	β	β	β	β	β
Subject involvement on household chores	0.09	0.15	−0.09	−0.20^∗∗^	−0.10	−0.11
Perception of partner involvement on household chores	0.12^∗^	0.09	0.08	0.01	0.08	0.23^∗^
Marital conflict	0.42^∗∗^	0.02	0.22^∗∗^	0.11	0.26^∗^	−0.03
	*R*^2^ = 0.18^∗∗^	*R*^2^ = 01	*R*^2^ = 0.06^∗∗^	*R*^2^ = 0.02	*R*^2^ = 0.07^∗∗^	*R*^2^ = 0.06^∗^

*^∗∗^p < 0.01, ^∗^p < 0.05. Standardized beta coefficients (N = 515).*

ANOVAs results confirm partially hypothesis 3 since greater involvement of women in household chores do not generate a statistically significant increase in WFC comparing to men. There are gender differences in the extent to which this differential involvement in domestic tasks affects FC and (in a tangentially significant way) WC that point to a gender effect. On one hand, in the case of women, when their involvement in household chores is high, their FC and WC levels are similar; however, when their involvement is low, FC decreases and WC increases. On the other hand, in the case of men, the WC is always greater than the FC regardless of their degree of involvement in household chores. That is, in the case of women when there is a lower involvement in household chores the FC is also lower, but increases the WC.

There are no gender differences regarding the WFC according to the perception of their partners: it increases significantly in both men and women when the involvement in household chores of the partner is high or low, being always higher among women than among men regardless of the involvement of the partner with household chores is high or low, which completely rejects hypothesis 3.

It is noteworthy that the effect of the perception of involvement of the partner in household chores by gender does not affect WC or FC in a gender-specific way, but it affects the WFC globally statistically significantly, although these differences were not gender effects manifest. This indicates that the WFC is affected by the involvement of the partner in household chores, but not for the involvement of the subject in them, which segmentally would affect the FC and WC.

Regarding hypothesis 4, the increase of conflict by domestic tasks among the partners does not affect the WFC in a statistically significant way in women nor in men, but it does on WC and FC: when MC is high WC increase both in women and men, but FC increase only in women.

As a confirmation of this results, regarding the relationship between the subject’s and partner’s involvement in household chores and the different conflicts, regression analyses (see **Table 5**) show, first, that subject involvement on household chores does not predict WFC in women nor men, but only WC in men in a negative way. Moreover, the perception of the partner’s involvement in household chores and MC is a predictor of women’s WC and men’s WFC. Again these results do not confirm hypothesis 3.

Nevertheless, regarding hypothesis 4, as a difference of the ANOVA results, the increase of conflict by domestic tasks among the partners predict the WFC, WC, and FC in a statistically significant way in women but not in men. So results show that MC in women predicts WFC. This result fully support hypothesis 4. In addition to this, the MC is the only variable of those studied that affects the FC in the case of women, whereas involvement in housework does in the case of men, supporting also hypothesis 4.

In the case of men, the perception of the partner’s (women) involvement in household chores is a predictor of WFC. Results also show that men’s involvement in household chores is a negative statistically significant predictor FC as their beta coefficient is negative. That is, it seems that when the involvement of men in housework increases, the conflict in the family decreases; but when the perception of involvement of their female partners is high, it increases in them the WFC. However, MC does not predict this FC in men, so the FC does not increase by the conflict with the partner for housework but by their low involvement in them.

## Discussion

Home-work interaction has been the focus of a wide range of scientific literature during the past decades. It is generally accepted that both the family and the work scope affect each other in a different way. However, it was not studied in which degree the own and the partner’s involvement in family issues affect different kind of work-home conflict from a gender point of view. Thus, the aim of this study was to check whether the unequal involvement in household chores between men and women is associated with increased WFC in women, and explain it in terms integrating the knowledge of gender studies.

First, results confirm inequality because it indicates that the involvement of women in household chores is, on average, more than double the involvement of their male partners. In addition, men are more involved in traditionally masculine household chores (i.e., home repairs and family management), and women are more involved in traditionally feminine chores (i.e., childcare or shopping). Symmetrically, the subject’s perception of the partner implication confirms this difference: women perception of their men partner involvement in household chores much less than men perception of their woman partner involvement. Therefore, hypotheses 1 and 2 of our study are confirmed.

Secondly, we checked if those unequal involvements relate differently to men and women on different ways of WF interaction. We found that the greater involvement of women in household chores does not affect the level of WFC differentially in men and women, so hypothesis 3 is not met. This gender inequality in the distribution of household chores and child care does not imply a higher level of WFC in women compared to men. Rather the opposite happens: when more involved are both men and women in household chores, lower is the WFC. Although the hypothesis 3 is not corroborated, it should be noted that when the involvement of women in household chores is high, their level of FC increases; when men’s involvement increases, their level of WC increases, which in some way supports hypothesis 3. That is, the high involvement in household chores has negative consequences in the family sphere for women and in the workplace for men, possibly because of the greater respective importance that women give to family and men to work, as it poses the traditional gender role model.

In addition to this, results show that when the involvement of women in household chores is high, their levels of WC and FC are similar, i.e., it equally affects both areas. When this involvement is low, FC is lower than the WC. However, among men, WC is always greater than the WC regardless of their involvement in household chores. Furthermore, when the conflict with the partner for household chores is high, women report a higher FC but not a higher WC, whereas in man this conflict does not affect neither the FC nor the WC.

However, in the case of women, MC affects conflict related WC and FC and WFC, so hypothesis 4 is fully corroborated. This is very interesting because although hypothesis 3 is not met, however, the conflict with the partner due to this inequality in the distribution of housework seems to generate this WFC. That is, it would not be the greatest involvement in household chores itself that might cause and increase WFC in women, but the conflict with their partner which might produce it.

These results may be related to the absence of perception of injustice in the relationships regarding to inequality in the distribution of domestic and family responsibilities between men and women, so that in many cases women neither do perceive injustice in their relationships nor are dissatisfied. Following the review of Yago and Martínez (2009), it has repeatedly shown that the perception of an unequal distribution of housework between men and women does not necessarily lead to a perception of unfairness. This perception of justice on the division of domestic work and the ideology of traditional gender that supports it explain why gender inequalities remain in the family sphere mediating the relationship between the perception of injustice and perceived quality the relationship. In fact, when women are more socially and emotionally independent from their partners, they are more likely to consider unfair the inequality in the distribution of household chores.

The perception of injustice is a mediating factor between an unequal distribution of domestic work and the perceived quality of the relationship; the relationship may be perceived as satisfactory although the sharing of responsibilities is not equal, if it is not perceived unfair (Yago and Martínez, 2009). However, these results were mediated by gender ideology so this inequal distribution do not generate distress in the more traditional women whereas it does in women with an equal gender ideology.

In this line a study of Ogolsky et al. (2014) shows that the discrepancies at a cognitive level between men and women with regard to equality in household chores affects the quality of the relationship in the sphere of the couple in greater way to women than in men. However, when this inequality is manifested in a behavioral level, it does not seem to affect the quality of the relationship in women. That is, the real inequality does not affect the quality of the relationship in women, but it does at the cognitive level.

The involvement of the couple in household chores is related to an increased WFC, although it does not affect the WC or the FC separately by gender, but affects the WFC globally: it increases similarly in men and women when the couple’s involvement is high. This indicates that the WFC is affected by the involvement of the partner in household chores, but not for the involvement of the subject in them, which would affect to a segmented FC and WC. These results do not prove the hypothesis 3, but can indicate that the model of traditional gender roles does not serve to satisfactorily explain the influence of the division of household tasks and the effect of gender inequality in the WFC, as both in the case of men and women more involved in household chores generate that their female and male partners feel an increased WFC.

Men’s and women’s perceptions of their partners’ involvement in household chores contribute significantly to the perception of WFC; their own involvement also contributes significantly to FC, but negatively, which means that the more involved their partner is in the household chores, the greater their WFC.

Although our study seems to show that gender is an important variable in the involvement in household chores, and that gender inequality and the model of traditional gender roles is still valid in our western society, it also seems to suggest that increased WFC due to a high involvement in household chores is not exclusive to men but also occurs in women. This could be an indicator of a change in the model of traditional gender roles that began in the 80s, where new generations equate the importance of work and family spheres in the cases of both men and women.

In fact, results of some recent research (Shockley et al., 2017) indicate that men and women appear to be more similar than different in their WFC experiences; gender differences in WFC appear to generally be small, regardless of which specific subgroups are examined, and when there is meaningful variation in the magnitude of gender differences in WFC the key factors that determine this variation is currently not well understood.

From this point of view, several alternative models other than the conflict perspective might explain these results. This tis the case of models such as the synergy between work and family, positive balance, work-family facilitation, or work-family enrichment (Beutell and Wittig-Berman, 2008; Lapierre et al., 2017), which would better understand the effect of gender on the individual’s relationship between work and family.

The use of this new model integrative approach is justified by the social changes that characterize the values of the new generations, Gen Xers (born between 80 and 2000 population). They seem to consider that both work and family are equally important in their life, and try to find the most appropriate way to reconcile both aspects (Beutell and Wittig-Berman, 2008), giving less importance to presentism at work and being supporters of flexibility. This understanding of the work is based, in addition to the facilities provided the digital revolution and technologies for work, making workers less dependent of a particular physical space and a fixed schedule to perform their work, together with the values of personal autonomy and responsibility that are shared by this new generation. This facilitates that people can now have more time to devote to other areas of their life within the scope of non-work such as family or leisure, with a progressively greater importance in their social identity.

From this point of view, research on work and family interaction has evolved from the study of isolated variables within the conflict and segmentation models toward more complex models that try to understand from the boundary theory, and the models of facilitation and synergy, how transitions are made from one scope to the other, and how they integrate with each other. They do not consider them as separate domains but as something unitary and unbreakable within the life of people. In the same way, an approach that takes into account the gender ideology is progressively being imposed, since it is inseparable from the relationship between work and the family from a cultural point of view.

### Study Limitations

This study focuses on the effect of different kinds of conflict related to the home and work settings. However, due the lack of clear differences in results regarding WFC in men and women when partners’ implication in household chorus is high, it would be necessary to include facilitation and synergy models that would make it easier to understand the work-family relationship in all its facets, including the role played by gender and gender inequality. Research on the positive reciprocal effects of work and family is fundamental to understanding the complexity of the work-family interaction.

In addition, this study has other methodological limitations. First, we studied the effect of gender and involvement in household chores on the work-family relationship using independent samples of men and women, without collecting data from their partners. However, we analyzed the perceptions of these people (men and women) about their own involvement and their partner’s involvement, and this perception was shown to be significant. Nevertheless, it would be interesting to include the whole couple as a unit in future studies to increase the reliability of the proposed model.

Second, this study is based only on quantitative analyses. It would be interesting to support these results with qualitative studies (through interviews or focus groups) that would help us to interpret the analyses of the results framed in both the traditional gender roles and cross-effect theories, but also in people’s interpretations, increasing the model’s validity. They would also allow us to understand the gender role in the direction of the cross-effects of work stress from men to women, or from women to men, as our results only partially support this cross-effect, contrary to previous results (Bakker et al., 2008). In any case, the quantitative methodology used in this study allowed us to detect, in a relatively simple way, the existence of changes in the relationship between gender and the traditional division of roles as a first step.

Also, the household chores used are those that might be generalized to mostly couples with children at school age. However, we have not considered specific situations (i.e., living in their house, living in a large or in a small town, grandparents support in caring children, age of the children) that might have help us to better describe the sample and interpret our results. Future studies could include this kind of sociodemographic variables.

In addition, may be other methodological limitations that may have conditioned the results. One of them is the imbalance in the percentage of men (63%) regarding women (37%). However, this limitation is assumable given the correlational nature of the study and the breadth of the sample. Finally, the reliably of the involvement of the partner in household chores is not too high (Cronbach’s alpha 0.62) which could raise doubts about its effect as an independent variable in the WFC in men and WC among women. Nevertheless, it met widely accepted criteria to assume its reliably (over 0.60).

### Practical Implications

These results raise a number of practical implications for equality between men and women in terms of gender issues in the effective management of organizations in order to establish social integration and equality policies in both family and work settings (Wharton, 2015). The management of work and working time within organizations must take into account the social changes occurring in gender roles, and start to consider that both men and women gradually tend to give the same importance to their work and family environments (Kuo et al., 2018), with the accompanying increase in WFC and stress in both partners. Thus, although in many cases traditional gender roles are still valid (the family sphere continues to be more important for women than for men), it is necessary to consider the vision and specific attitudes that both workers have about their involvement in work and family, and establish organizational policies that help to reconcile both spheres in both genders (Lucas-Thompson and Goldberg, 2015).

Moreover, public and social institutions specializing in family matters should incorporate these progressive changes in traditional gender roles into their strategies, in order to facilitate the homogenization of women’s and men’s roles within the family and workplace. For instance, they can design family counseling and couple training campaigns that help them to discover how to best coordinate their dedication to the family in a way that will reduce stress and conflict, and how to minimize WFC, even translating it into work-family synergy.

But also organizations might participate in this social change. They might contribute for instance through the inclusion of family friendly politics to support the search for home-work balance of their workers, men and women (Sprung et al., 2015; Lin et al., 2017; Matias et al., 2017). It would mean a way to improve the quality of working life of their workers and, at the same time, a return of investment (ROI) both for the organization (Dowd et al., 2017) and for our, hopefully, every time more equitable society.

## Ethics Statement

All participants provided written informed consents before to complete the survey, in accordance with the Declaration of Helsinki, and researchers guaranteed the anonymity of data. This study was approved by the institutional review board of the Faculty of Labour Relations and Social Work of the University of Basque Country.

## Author Contributions

JC has been the director of review of the scientific literature, theoretical justification, methodology design, data collection, statistical analyses, and results description. EC has coordinated the improvement of the whole design and redaction paper, including conclusions and research limitations.

## Conflict of Interest Statement

The authors declare that the research was conducted in the absence of any commercial or financial relationships that could be construed as a potential conflict of interest.
